# Time-dependent efficacy and safety of tooth bleaching with cold plasma and H_2_O_2_ gel

**DOI:** 10.1186/s12903-022-02601-8

**Published:** 2022-11-24

**Authors:** Xiaohui Yang, Ke Sun, Weidong Zhu, Yinglong Li, Jie Pan

**Affiliations:** 1 Department of Stomatology, Children’s Hospital of Soochow University, Suzhou, 215025 China; 2 Department of Cariology and Endodontology, Qingdao Stomatological Hospital Affiliated to Qingdao University, Qingdao, 266001 China; 3 Department of Applied Science and Technology, Saint Peter’s University, Jersey City, NJ 07306 USA; 4 Department of Stomatology, Beijing Chaoyang Hospital, Capital Medical University, Beijing, 100020 China; 5 Department of General Dentistry, Peking University School and Hospital of Stomatology and National Clinical Research Center for Oral Diseases and National Engineering Laboratory for Digital and Material Technology of Stomatology and Beijing Key Laboratory of Digital Stomatology, Beijing, 100871 China

**Keywords:** Cold plasma, Tooth bleaching, Mechanical safety, Pulp chamber temperature

## Abstract

**Background:**

Hydrogen peroxide (H_2_O_2_) is the commonly used bleaching agent for teeth. But it is highly corrosive to teeth for the high concentration. The cold atmospheric pressure plasma has been witnessed a novel tooth bleaching technology and could help strengthen the bleaching effect when combined with H_2_O_2_. However, the efficacy and safety might highly correlated with processing time. The present study aims to evaluate the time-dependent efficacy and safety of tooth bleaching with cold plasma and H_2_O_2_ gel in vitro.

**Methods:**

The H_2_O_2_ concentrations of the gel used in the study are 6%, 15%, 25% and 35%, respectively and the treatment time varies from 5 to 20 min. The tooth bleaching effect was evaluated by a Crystaleye Spectrophotometer and the overall change of the colorimetric value based on three independent measurements. Meanwhile, the microhardness, roughness and tooth temperature were evaluated. The surface morphology and the elemental composition were determined by scanning electron microscope and energy-dispersive X-ray spectroscopy.

**Results:**

5 min bleaching treatment contributed to 60% of the bleaching effect maximum, the 10 min effect was close to 15 min effect. Meanwhile, the microhardness reduced and roughness increased under a treatment which was longer than 20 min. Tooth pulp chamber temperature was keeping in a safe range within 20 min treatment.

**Conclusion:**

5–10 min was the best treatment time from which we can get an ideal tooth bleaching effect and less influence on tooth enamel and pulp tissue when using cold plasma and H_2_O_2_ gel.

## Background

Tooth bleaching has become one of dentistry’s most popular esthetic services as it is the most conservative treatment for discolored teeth [1, 2]. The bleaching of teeth using peroxide is now widely recognized as a safe and effective method for tooth bleaching and has become a routine dental procedure [3, 4]. However, high concentration of H_2_O_2_ and prolonged treatment are potential obstacles for the wide adoption of tooth bleaching [5–7]. Infrared CO_2_ laser and LED can improve bleaching efficacy but the heat released during the process has raised much apprehension in patients [8].

Plasmas are often referred to as the fourth state of matter after the commonly seen three states: solid, liquid and gas. They can in general be considered as gases with certain degree of ionization, and collectively respond to external electric and magnetic fields. Plasmas are abundant in nature and comprise 99% of the visible universe. They can be roughly categorized into “hot” and “cold” plasmas. The “hot” plasmas, with the overall gas temperature ranging from 10^4^ to 10^7^ K, are often used in applications such as cutting, welding and fusion. The overall gas temperature of the cold plasmas is much lower, usually close to room temperature. They are often generated at low gas pressures and are used in areas such as surface modification, thin film deposition and lighting. Cold plasmas being generated at atmospheric pressure is a more recent endeavor, owing to the clever designs of the devices and the development of power sources.

It is reported that the combination of bleaching gel and plasma can significantly improve the bleaching efficacy, comparing to using bleaching gel alone for the same amount of treatment time [9–13]. During the bleaching process, ultraviolet radiation, heat and reactive species such as hydroxyl radical, superoxide anion and singlet oxygen interact directly or indirectly with the enamel and the pulp tissue, leading to teeth pulp chamber temperature increase, enamel surface morphology and microhardness modification [14]. One needs to address some safety concerns before this technology can eventually be adopted in dental clinics.

It is hypothesized that the efficacy of tooth bleaching is positively correlated with cold plasma treatment time. Thus, this in vitro study aims to investigate how the cold plasma treatment time affects the bleaching efficacy when bleaching gels containing 6%, 15%, 25% and 35% H_2_O_2_ are used in the experiments. The change of enamel surface microhardness and morphology, as well as the pulp chamber temperature are monitored.

## Methods

### Tooth selection and sample preparation

**Fig. 1:**
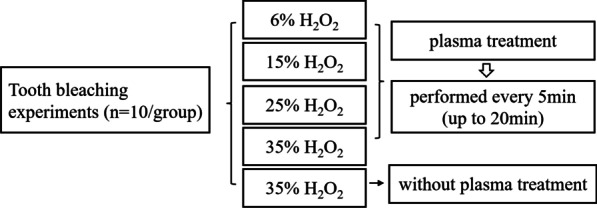
Flowchart of tooth bleaching experiment

Extracted human teeth with intact crowns, free from caries, crack or other defects were collected, a preliminary screening was performed at the time of selection to avoid obvious color difference. Fifty teeth were randomly divided as five groups (n = 10/group): 6% H_2_O_2_ with plasma, 15% H_2_O_2_ with plasma, 25% H_2_O_2_ with plasma, 35% H_2_O_2_ with plasma (referred to as the “plasma groups”) and the H_2_O_2_ group (negative control group). The sample size (n = 10/group) was determined with reference to our previous studies [11, 15]. The overall flowchart of tooth bleaching is shown in Fig. 1.

Upon various treatments, thirty-six tooth pieces with an enamel area of 3 × 3 mm^2^ were prepared and used to analyze microhardness and roughness. Besides, extra nine caries-free, unrestored human premolar, central incisor teeth were used to monitor the tooth pulp chamber temperature. The apical part of the root of these nine teeth was cut (about 2–3 mm) to the cementoenamel junction (CEJ), and the apical orifice of the root canal was enlarged by size #1 round bur. The remaining pulp tissue was removed from the canal, and the sample was cleaned and stored in 0.1% Thymol solution at 4 °C before the experiments.

### Cold plasma device

**Fig. 2:**
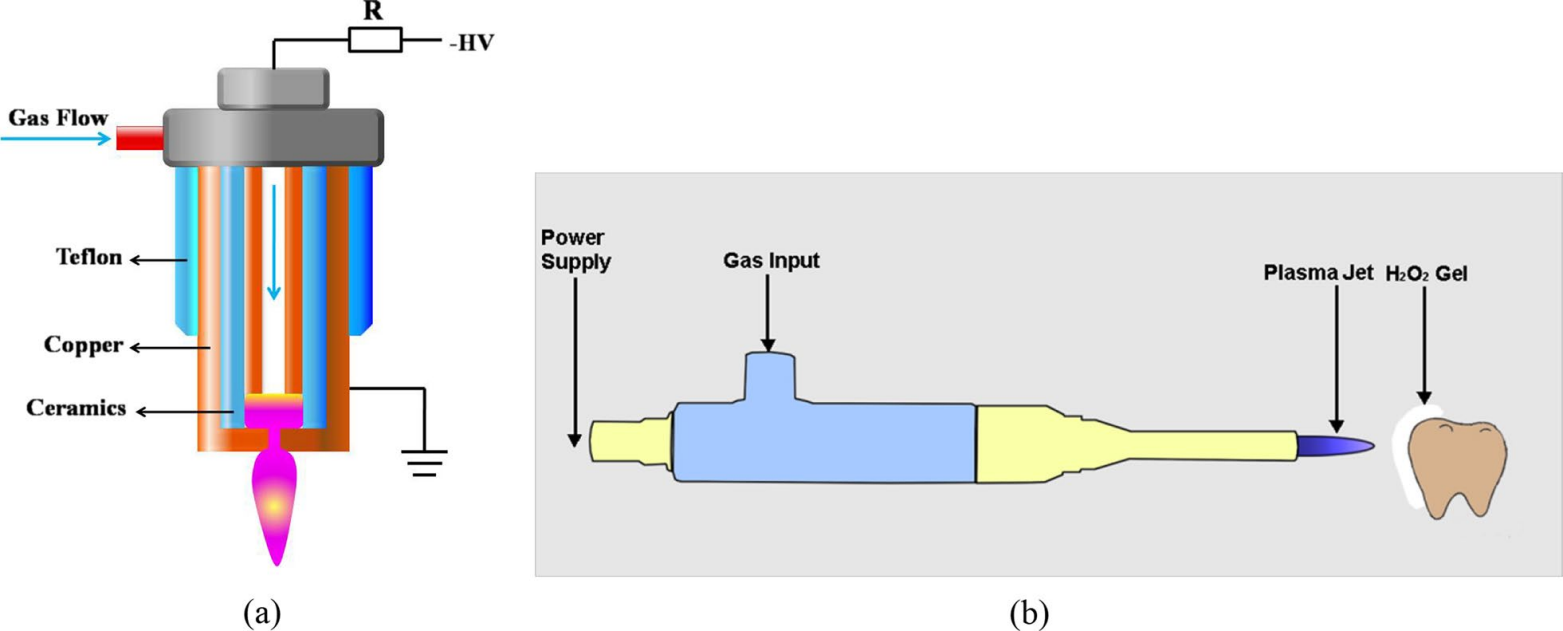
**a** A schematic diagram of the plasma device and the PMJ. **b** Schematic diagram of the PMJ treatment of extracted tooth (clinical H_2_O_2_ gel was applied to the tooth surface every 30 s).

The major section of the plasma device used in this study was comprised of two copper tubings and a ceramic tubing as an insulator spacer between them (as shown schematically in Fig. 2). All three tubings are arranged concentrically. A copper end cap with a circular hole of the same size as the inner diameter of the inner copper tubing (~ 0.8 mm) was attached to the outer copper tubing, setting the inter-electrode distance to ~ 0.5 mm. Negative polarity direct current high voltage (~ 0.6 kV) was applied to the inner electrode while the outer electrode was grounded for safety purposes. Compressed air at a flow rate of ~ 5 SLPM (standard liters per min) was used as the working gas and pushed the plasma out of the hole on the end cap, forming a plasma microjet (PMJ) of ~ 1 cm in length [11]. The whole device is ~ 15 cm long and weighs less than 200 g and the tip of the plasma micro-jet can be safely touched human finger without any discomfort.

### Tooth bleaching experiments

As Fig. 1 shows, fifty teeth were randomly divided into five groups (n = 10). Teeth in the control group were covered ~ 1 mm thick 35% H_2_O_2_ gel and were not subjected to any plasma exposure. Teeth in the other four groups received 6%, 15%, 25% and 35% H_2_O_2_, respectively and in the meantime were treated with the plasma every 5 min until up to 20 min. In order to ensure an adequate amount of gel on the surface of tooth throughout the treatment period, the gel was replenished at an interval of 30 s. The distance from the exit nozzle of the plasma device to the tooth surface was kept at 1 cm. Upon completion of the plasma treatment, the residual gel was wiped off the teeth with Kimwipes. The color of the teeth were evaluated by a Crystaleye Spectrophotometer (Olympus Corporation, Tokyo, Japan) and the overall change of the colorimetric value based on three independent measurements was expressed in the Commission Internationale de L'Eclairage (CIE) L^*^a^*^b^*^, or CIELAB Color Scale following formula: $\Delta E^{*} = \sqrt {(\Delta L^{*} )^{2} + (\Delta a^{*} )^{2} + (\Delta b^{*} )^{2} }$.

### Microhardness and roughness test

Microhardness and surface roughness are the major indexes to evaluate the performance of tooth hard tissue, and pulp chamber temperature is an important factor of pulp irritation. Extracted human teeth were cut longitudinally into bar-shape test specimens and embedded in epoxy resins with a surface area of 3 mm × 3 mm exposed. A total of 36 test specimens were randomly divided into six groups (N = 6): a blank group (without any treatment), a negative control group (35% H_2_O_2_ alone), and four plasma groups with various concentrations of H_2_O_2_ as aforementioned. In order to keep the baselines of the microhardness and the surface roughness measurements at the same level, the specimens were polished before the treatments, following a sequential manual polishing protocol with 800#, 1000#, and 1200# water-proof abrasive papers. The final step of the polishing was done with a polishing cloth and multicrystalline diamond abrasive paste on a polishing machine (p-1 metallographic specimen cutting machine, Laizhou Weiyi Testing Apparatus Manufacture Co., Ltd, China). Microhardness and roughness measurements of the enamel were performed before and after the plasma or pure H_2_O_2_ treatment. The microhardness was measured three times for each specimen using a Shimadzu HMV tester with a Knoop indenter at a load of 0.9807 N for 15 s [12]. The surface roughness was measured three times for each specimen using a Mitutoyo SJ-400 portable digital roughness tester with 3 steps, 0.08 mm per step. The mean values of the change in microhardness and surface roughness from before to after the treatments were evaluated with the analysis of variance (ANOVA) method via SPSS (Statistical Package for the Social Sciences. IBM Corp., V14) and changes were considered significant, if *p* < 0.05.

### SEM and EDX

Third molar teeth were chosen, and each tooth was longitudinally cut into four bar-shaped test specimens with a surface area of 3 mm × 3 mm. Specimens were randomly divided into five groups (n = 3/group): none treatment group (0 min), tooth bleaching assisted by PMJ with 35% H_2_O_2_ for 5 min, 10 min, 15 min and 20 min. The gel on tooth surfaces of the specimens were slightly cleaned and dried at 37 °C overnight before test. In an effort to achieve better electrical conduction of the samples, the sample surface was sputter-coated with a 20-nm gold film. A low deposition rate and sufficient target-to-sample distance were chosen with substrate cooled in order to avoid sample damages during the sputtering process. The surface morphology and the elemental composition of each sample was determined by scanning electron microscope (SEM, Hitachi S-4800, Japan) and energy-dispersive X-ray spectroscopy (EDX, AXIS Ultra, Kratos, England). The differences in the atomic abundance of O, Ca, and P among various treated samples were statistically analyzed with ANOVA. The changes were considered significant when *p* < 0.05.

### Tooth pulp chamber temperature monitor

**Fig. 3:**
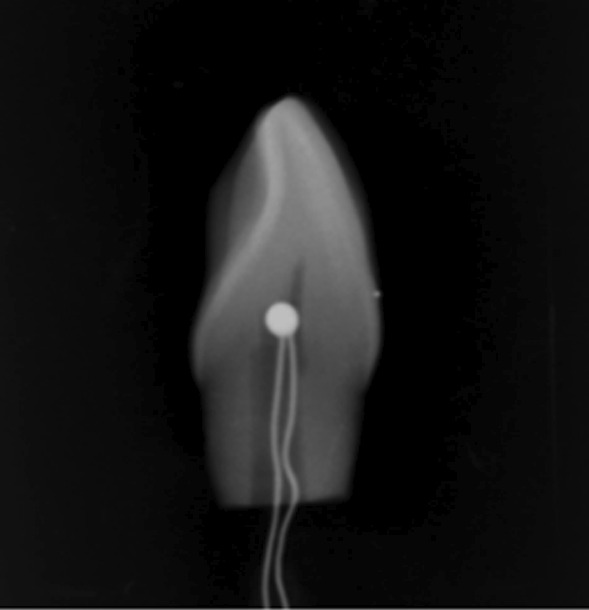
A digital radiographic image obtained to confirm the position of the thermocouple

As Fig. 3 shows, a digital thermometer (K-type thermocouple) (Shenzhen Victory electrical technology, Guangzhou, China) was used to monitor the temperature of tooth pulp chamber during a 20 min plasma tooth bleaching treatment. The empty pulp chamber was filled with heat sink compound, and then inserted K-type thermocouple into it through the cut root area. The thermocouple was placed at the uppermost coronal level of the pulp chamber, and the temperatures were recorded every 30 s. A digital radiographic image was obtained to confirm the position of the thermocouple.

## Results

### Effect of treatment time on tooth bleaching efficacy

**Fig. 4:**
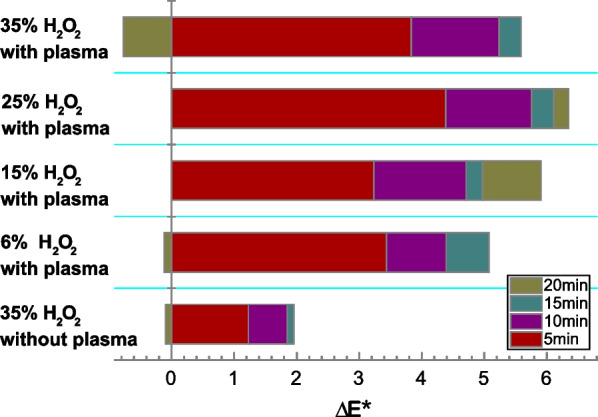
The color change of different group in each period, the positive direction means increase and the negative direction means decrease

Figure 4 shows how every 5 min treatment (up to 20 min) contributes to the change of colorimetric value ΔE* of the teeth. About 65% of the maximum color change in each case was attributed to the first 5 min treatment. About 90% of the maximum color change was attributed to the first 10-min treatment, including the negative control group. A 5 min further treatment leads to about 8–10% further improvement of the color change. The contribution to the color change in general decreases with every 5 min further treatment. The 15% H_2_O_2_ and plasma group behaved slightly differently, with the last 5-min treatment contributing more than the previous 5 min. It is interesting to note that a color regression was seen in some cases, especially the 35% H_2_O_2_ with plasma group. The 6% H_2_O_2_ with plasma group and 35% H_2_O_2_ alone group also showed color regression but not as prominent.

In the clinics, a ΔE* > 3.0 means a visible color change and a ΔE* > 3.7 corresponds to a significant color change [16]. The numbers and percentages of teeth resulted in ΔE* > 3.0 and ΔE* > 3.7 in the plasma groups and in the control group are summarized in Table 1. In the plasma groups (including all concentrations of H_2_O_2_ tested), 55% of the samples reached ΔE* > 3.0 in the first 5 min treatment, followed by 85%, 87.5% and 90% of the teeth reaching ΔE* > 3.0 with an additional 5, 10 and 15 min treatment, respectively. 10% of the teeth in the plasma groups, however, showed color regression, which means they cannot reach ΔE* > 3.0 even after a 20 min treatment. The percentages of teeth reaching ΔE* > 3.7 after each 5 min treatment are slightly lower than the ΔE* > 3.0 case as expected. Nevertheless, 80% of the tested teeth reached ΔE* > 3.7 with a 10 min treatment. Additional 5 to 10 min treatment only resulted in a slight increase the percentage to 82.5%. Meanwhile, only 10% of the teeth in the control group reached ΔE* > 3.0 after the first 5 min treatment. 5 and 10 min additional treatment led to 20% of the teeth reaching ΔE* > 3.0. The percentage dropped down to 10% after a 20 min treatment due to an obvious color regression.

**Table 1:** Numbers and percentages of teeth resulted in ΔE* > 3.0 and ΔE* > 3.7 in the plasma groups (including all concentrations of H_2_O_2_ tested) and the control group after 5, 10, 15 and 20 min treatment, respectively

Treatment time (min)		5		10		15		20	
Number and percentage		n	%	n	%	n	%	n	%
6%H_2_O_2_ with Plasma Groups	ΔE* > 3.0	5	50	8	80	9	90	8	80
	ΔE* > 3.7	3	30	8	80	9	90	8	80
15%H_2_O_2_ with Plasma Groups	ΔE* > 3.0	4	40	9	90	7	70	9	90
	ΔE* > 3.7	4	40	9	90	7	70	9	90
25%H_2_O_2_ with Plasma Groups	ΔE* > 3.0	7	70	9	90	10	100	10	100
	ΔE* > 3.7	5	50	9	90	9	90	9	90
35%H_2_O_2_ with Plasma Groups	ΔE* > 3.0	6	60	8	80	9	90	9	90
	ΔE* > 3.7	4	40	6	60	8	80	7	70
Control Group	ΔE* > 3.0	1	10	2	20	2	20	1	10
	ΔE* > 3.7	0	0	1	10	1	10	1	10

### Microhardness measurement

**Fig. 5:**
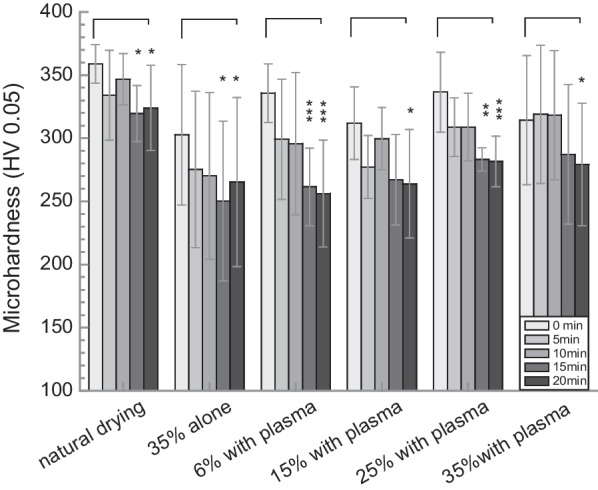
The enamel microhardness in each period of various groups treated up to 20 min

The enamel microhardness (HV) of teeth treated with 35% H_2_O_2_ alone and with 6–35% H_2_O_2_ with the assistance of plasma were recorded up to 20 min and are plotted in Fig. 5. Also included in Fig. 5 are microhardness data from naturally dried teeth. The microhardness of teeth in general decreases with the increase of treatment time. Statistical analysis shows there are significant microhardness change occurred between 0 and 15 min, 20 min of each group, and no significant difference in each period among various groups.

### Surface roughness measurement

**Fig. 6:**
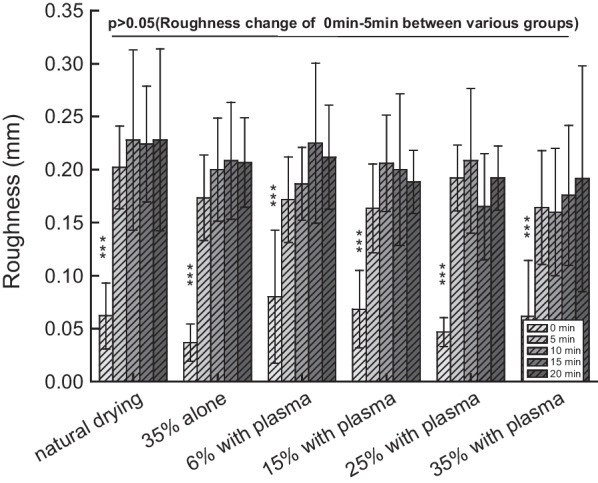
The enamel surface roughness in each period of various groups treated up to 20 min

Enamel surface roughness of teeth in different groups treated up to 20 min was evaluated and plotted in Fig. 6. An abrupt change of roughness was observed in the first 5 min, including the naturally drying samples. Subsequent roughness changes are less significant and are within the error range. The trend of the plasma groups is similar to the blank group and the negative control group. Statistical analysis shows there is a significant roughness change occurred in the first 5 min of each group, and no significant difference which exhibited in the first 5 min between the natural drying group and each treatment group.

### Tooth morphology and chemical composition

**Fig. 7:**
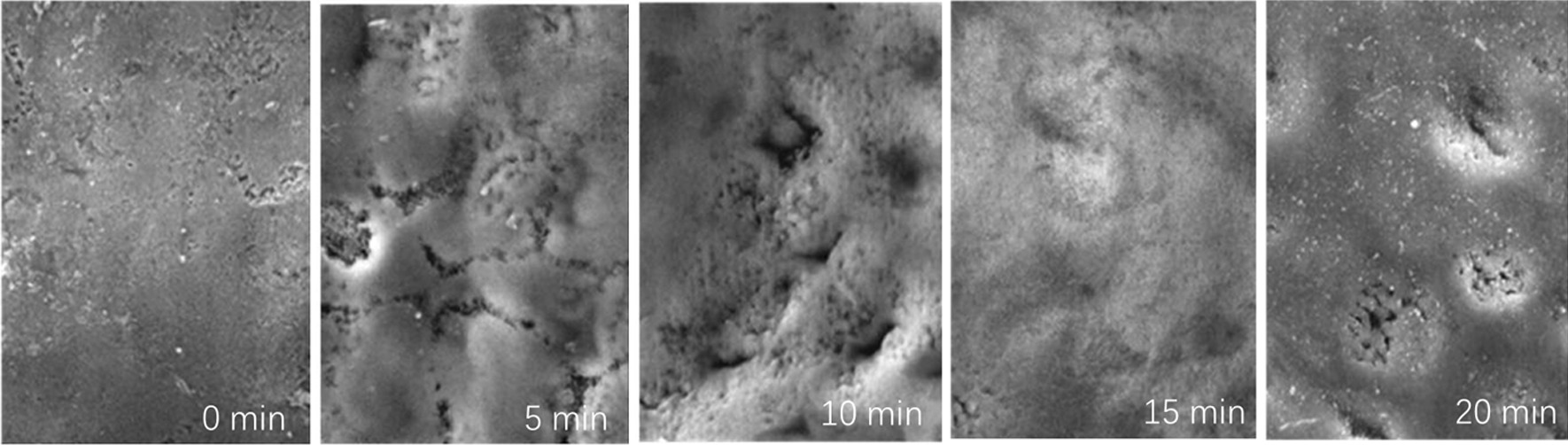
The morphology of tooth enamel surface after tooth whitening by plasma with 35% H_2_O_2_ for 0 min to 20 min (×5000 magnification)

**Fig. 8:**
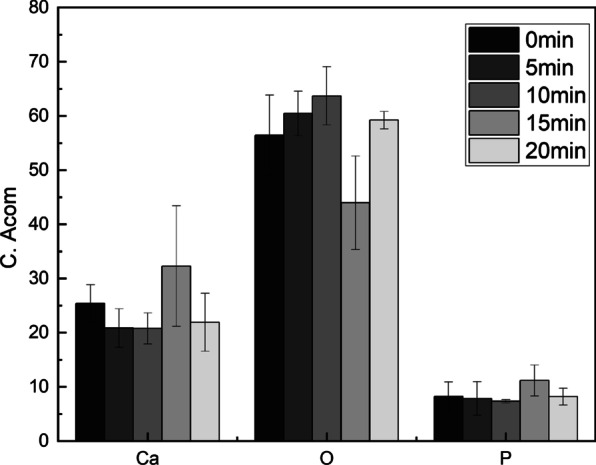
The element composition of enamel surface without treatment (0 min) and after tooth whitening by plasma with 35% H_2_O_2_ for 5–20 min

Since the 35% H_2_O_2_ with plasma group showed the most obvious color regression (Fig. 4), we chose to analyze the morphological and chemical composition change (compared with 0 min) of this group after 5 min, 10 min, 15 min and 20 min treatment. As Fig. 7 shows, doughnut-shaped erosions appear after 5 min treatment. The enamel rod melted from center first and expanded to the surrounding enamel with further treatment time (15 min). A 20 min treatment led to a sooth enamel surface in which the surrounding enamel melted to a level much similar to the center of enamel rod, resulting in a morphological appearance much like the untreated enamel. This result is consistent with roughness result in Fig. 6. Meanwhile, as Fig. 8 shows, the atom amounts of O, Ca, P of the teeth in this group showed a significant change after a 15 min treatment, but returned to a level similar to those at 5 and 10 min with an additional 5 min treatment.

### Tooth pulp chamber temperature

**Fig. 9:**
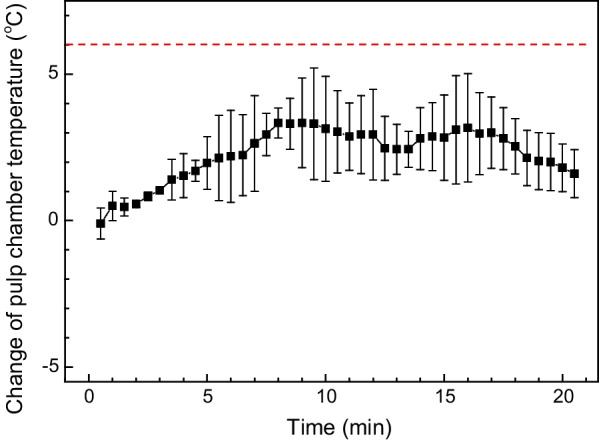
The change of tooth pulp chamber temperature during a 20 min treatment with 35% H_2_O_2_ assisted by plasma

The time evolution of the tooth pulp chamber temperature change (*ΔT*) is recorded in the 35% H_2_O_2_ with plasma group and plotted in Fig. 9. The initial room temperature is ~ 25 °C and the maximum *ΔT* is approximately 4 °C during the 20 min treatment. Temperature changes at other H_2_O_2_ concentrations were not recorded but we do not expect much deviation from what is reported here.

## Discussion

The present study indicated that tooth bleaching efficacy is partly positively correlated with the treatment time, prolonging the treatment time may lead to color regression, which is contrary to our hypothesis. There are essentially two factors that are of concern: (1) the concentration of H_2_O_2_ in the dental gel, (2) the total time of the procedure. Previous studies showed that with the assistance of cold plasma, even low concentrations of H_2_O_2_ (down to 6%) can result in an excellent tooth bleaching efficacy [11] without an unacceptable damage on tooth enamel [17]. Thus, this study focused on the operating time because opening mouth for long time is not only an additional load for the patients.

During the bleaching process, the appearance of the teeth is due to the complex interplay of the breaking of the long molecular chain of pigment stains, change of enamel surface roughness and enamel demineralization. A 5-min treatment results in 65% of the maximum color change. A 10-min treatment leads to approximately 90% of the maximum color change. A saturation of color change is likely achieved with a 15-min treatment. Further increase of treatment time may lead to color regression. This is in particular clear in the 35% H_2_O_2_ and plasma group. This finding is consistent with the so-called “saturation point” in teeth bleaching as reported in reference [18]. Beyond this “saturation point”, the porosity and brittleness of the enamel may increase (i.e. enamel damage) and the bleaching process must be stopped. The third 5-min treatment contributes little to the overall color change and may even be omitted in clinics.

In present study, 55% showed visible color change (ΔE* > 3.0) and 40% showed significant color change (ΔE* > 3.7) after a 5-min treatment. These percentages increase to 85% and 80%, respectively, after a 10 min treatment. There are, however, 10% of the teeth do not have visible color change even after 20 min treatment. This means combining H_2_O_2_ with cold plasma works majority of the time but is not effective for very single sample, as the bleaching efficacy depends on enamel and dentin structure of individual tooth and baseline of the tooth color to a certain extent.

The enamel microhardness in general decreases with the increase of treatment time, including in the naturally drying group and the negative control group. No significant differences on microhardness were observed within different treatment groups in each 5 min treatment period. On the other hand, enamel surface roughness in general increases with the increase of treatment time, with the first 5 min treatment contributing the most to the changes. These changes are attributed to the demineralization of enamel during the treatment process. The demineralization (erosion) patterns are related to the distribution of various enamel crystals (e.g. carbonated apatite, hydroxyapatite and fluorapatite) in the enamel rods (enamel prisms)[19]. The actual arrangement of the crystals in each enamel rod is rather complex. However, near the head of the enamel rod, the crystals are arranged parallel to the long axis of the rods. The central regions of the enamel rods are richer in carbonated apatite, which is more susceptible to acid demineralization than other crystallites found in enamel rods. Therefore, demineralization occurs preferentially in the central regions at the head of the enamel rods, leading to erosion patterns and thus rougher surface. The erosion then progresses along the central core, smoothing out the surface gradually, leading to the smoother surface at 20 min. This rough-smooth process repeat once the active agents been used.

No significant differences on surface roughness were observed within different treatment groups in each five-min treatment period, indicating the treatment time probably plays a more important role to demineralization than does the concentration of H_2_O_2_. The surface roughness results are further supported by the SEM data of teeth from the 35% H_2_O_2_ and plasma group: 5–15 min treatments result in surface roughness increase, while prolonged treatment of 20 min led to a smoother surface, which is similar to that of an untreated tooth surface. We believe this is actually caused by the extensive melt down of the enamel rods, which is completely different from the state of the teeth before the treatments.

Enamel is the hardest and most resilient tissue in the human body, which mainly composed of a hard mineral, carbonated hydroxyapatite (HA, Ca_10_ (PO_4_)_6_ (OH)_2_), packed at high density (95 wt% in mature enamel) [20, 21]. The main chemical elements are Ca, P and O. It is reported that HA is in contact with water, the following formula occurs [22]:

1 $${\text{Precipitation}} \rightleftarrows {\text{Dissolution}}$$

2 $${\text{Ca}}_{10} \left( {{\text{PO}}_{4} } \right)_{6} \left( {{\text{OH}}} \right)_{210} {\text{Ca}}_{2} \rightleftarrows 6{\text{PO}}_{{4}}^{{3{-}}} + \, 2{\text{OH}} -$$

3 $${\text{Solid}} \rightleftarrows {\text{Solution}}$$

A small amount of HA dissolves, releasing calcium, phosphate and hydroxyl ions. As Fig. 8 shows, although there is no significant difference between 0 min, 5 min, 10 min and 20 min (*p* > 0.05), the atom amounts of Ca and P decreased within 10 min, this is in accordance with formula. This indicated that demineralization occurs, which is also in accordance with SEM results. The O increased within 10 min mainly because that there are large amounts of ROS generated after cold plasma treatment. As treatment prolong to 15 min, the hydrogen ions has been removed: H^+^ + OH^−^ ⇄ H_2_O, so the O element decreased. However, the significant increase of Ca and P at 15 min are contrary to the formula. We speculate that enamel is mineralized layer by layer during growth and development, the new enamel surface explored after 15 min treatment.

Despite the similarity of the surface morphology, surface roughness and the atom amount of Ca, P and O between the teeth treated for 20 min and the untreated teeth, the microhardness is indeed different. This infers that teeth over-bleached may have a good appearance but the extensive melting of the enamel rods will eventually affect the strength of the teeth.

The tolerance of the temperature increase of pulp and bone tissue is limited. Zach and Cohen were the first to observe that a temperature increase of higher than 5.5 °C irreversibly damage the pulp tissue [18]. Other reports showed that the maximum pulp temperature increase is about 6 °C, and the acceptable value of bone temperature is below 42 °C [23, 24]. Several studies have proposed the use of thermocouples to evaluate tooth temperature directly during light irradiation for bleaching purpose [25–28]. K-type thermocouple was used in this study. The temperature increase was below 6 °C during a 20-min bleaching assisted by the plasma. This value is blow what was reported in our previous work [12], due to the fact that we measured the temperature change directly on teeth without applying dental gel. This value is also lower than that measured in LED bleaching for 30 s [24]. Other than mediating the reactive species between the plasma and the teeth surface, the dental gel may absorb the light to the tooth surface, and meanwhile serve as a good heat sink as it was replenished every 30 s during the treatment process, therefore reducing the internal pulp chamber temperature [29]. Actually, H_2_O_2_ gel was used to maintain humidness of tooth surface to avoid white plaque caused by dry and covered bleaching effect. The gel of a certain thickness can also buffer the directional heat irradiation from the plasma, averting local heating of the teeth.

## Conclusion

A cold plasma jet was applied to assist teeth bleaching by H_2_O_2_ of various concentrations. It takes only 5–10 min to achieve a satisfactory bleaching effect, with the enamel morphology, microhardness and surface roughness comparable to those from traditional clinical methods. Prolonged treatment to 20 min may lead to color regression as well as enamel damage. However, there are still some limitations, such as this is an in vitro study, the deep mechanisms of how cold plasma react with tooth enamel still unclear, the adhesion of microorganisms to the tooth surface has not been verified, the elements are involved in the enamel structure has still not been fully specified, the durability of the whitening effect still has not been verified.

## Data Availability

The datasets used and/or analysed during the current study are available from the corresponding author on reasonable request.

## Abbreviations

CAP: Cold atmospheric pressure plasma
H_2_O_2_: Hydrogen peroxide
CEJ: Cementoenamel junction
SLPM: Standard liters per min
PMJ: Plasma microjet
SEM: Scanning electron microscope
EDX: Energy-dispersive X-ray spectroscopy
